# Case-Crossover Analysis of Air Pollution Health Effects: A Systematic Review of Methodology and Application

**DOI:** 10.1289/ehp.0901485

**Published:** 2010-03-31

**Authors:** Eduardo Carracedo-Martínez, Margarita Taracido, Aurelio Tobias, Marc Saez, Adolfo Figueiras

**Affiliations:** 1 Department of Preventive Medicine and Public Health, University of Santiago de Compostela, Santiago de Compostela, Spain; 2 Santiago of Compostela Health Area, Galician Health Service [Servizo Galego de Saúde (SERGAS)], Santiago de Compostela, Spain; 3 Consortium for Biomedical Research in Epidemiology and Public Health [CIBER en Epidemiología y Salud Pública (CIBERESP)], Spain; 4 Institute of Environmental Analysis and Water Research [Instituto de Diagnóstico Ambiental y Estudios del Agua (IDAEA)], Spanish Scientific Research Council [Consejo Superior de Investigaciones Científicas (CSIC)], Barcelona, Spain; 5 Research Group on Statistics, Applied Economics and Health [Grup de Recerca en Estadística, Economia Aplicada i Salut (GRECS)], University of Girona, Girona, Spain

**Keywords:** air pollution, crossover studies, epidemiologic methods, health, systematic review

## Abstract

**Background:**

Case-crossover is one of the most used designs for analyzing the health-related effects of air pollution. Nevertheless, no one has reviewed its application and methodology in this context.

**Objective:**

We conducted a systematic review of case-crossover (CCO) designs used to study the relationship between air pollution and morbidity and mortality, from the standpoint of methodology and application.

**Data sources and extraction:**

A search was made of the MEDLINE and EMBASE databases. Reports were classified as methodologic or applied. From the latter, the following information was extracted: author, study location, year, type of population (general or patients), dependent variable(s), independent variable(s), type of CCO design, and whether effect modification was analyzed for variables at the individual level.

**Data synthesis:**

The review covered 105 reports that fulfilled the inclusion criteria. Of these, 24 addressed methodological aspects, and the remainder involved the design’s application. In the methodological reports, the designs that yielded the best results in simulation were symmetric bidirectional CCO and time-stratified CCO. Furthermore, we observed an increase across time in the use of certain CCO designs, mainly symmetric bidirectional and time-stratified CCO. The dependent variables most frequently analyzed were those relating to hospital morbidity; the pollutants most often studied were those linked to particulate matter. Among the CCO-application reports, 13.6% studied effect modification for variables at the individual level.

**Conclusions:**

The use of CCO designs has undergone considerable growth; the most widely used designs were those that yielded better results in simulation studies: symmetric bidirectional and time-stratified CCO. However, the advantages of CCO as a method of analysis of variables at the individual level are put to little use.

The first epidemiologic studies on the impact of air pollution on health were undertaken as a consequence of the extreme pollution episodes that took place in the decades from 1930 to 1960. The association between air pollution and certain health variables was made clear by simple graphic representations or by comparisons of mortality rates for these time periods (Firket 1931; Logan 1953). Since that time, air pollution levels have fallen substantially, such that, to evaluate their effects on health, longer time series are required. To this end, epidemiologists began to use dynamic regression models in the 1970s that consisted of models in which the relationship between the dependent and explanatory variables were distributed over time, rather than being expected to occur simultaneously. Moreover, investigators were able to control for residual autocorrelation, with the error being specified by means of autoregressive integrated moving-average models (ARIMA). The problem with these types of models is that they assume that the dependent variable is distributed normally, which, in fact, is extremely rare in the daily outcome count variables of morbidity and mortality events (Saez et al. 1999).

The early 1990s saw the appearance of linear models based on Poisson regression, in which a parametric approach was used to control for trend and seasonality because the event counts more typically have a Poisson distribution. These models use the variable “time” and its transforms, quadratic and sinusoidal functions (sine or cosine) of different frequency and amplitude, to control for the effect on the dependent variable (mortality or morbidity) of unmeasured variables that may vary seasonally, such as in pollen concentration, meteorological variables, and influenza outbreaks, or that may have a trend, such as changes in a city’s population distribution, in order to ascertain the effect of such variables on the dependent variable (Saez et al. 1999). Insofar as changes in a city’s population pyramid are concerned, Poisson regression is particularly useful only when cases, rather than the entire population, can be enumerated, because this form of regression analysis does not require knowledge of the denominator as long as population flux is in steady state (Loomis et al 2005).

Nevertheless, Poisson regression poses the problem that, if any of these unmeasured variables follows a cyclical component of varying frequency and width (as might be the case of pollen concentration or influenza), the parametric functions of time or of its sinusoidal transforms cannot be easily “adapted” to such changes. These limitations led to the development of nonparametric Poisson regression with the application of generalized additive models (GAMs) that use nonparametric functions of the variable “time” (Kelsall et al. 1997), which adapt flexibly to the irregular cyclic components of unmeasured variables and allow for flexible fits for important variables, such as temperature, barometric pressure, and relative humidity, thus reducing any potential confounding due to these factors.

One difficulty with this method is that the number of degrees of freedom of the smoothed nonparametric function must be specified by the researcher, with discrepancies arising as to the most appropriate way to calculate this. Because inappropriate determination of the number of degrees of freedom can lead to bias in the estimates of nonparametric Poisson designs, epidmiologists focused on the case-crossover (CCO) design that purported to control time trends. The CCO design was proposed by Maclure (1991) to identify risk factors of acute events; it is characterized by the fact that each subject serves as his or her own control by assessing referent exposure at a point in time prior to the event. By virtue of its design, this type of study controls for the influence of confounding variables that remain constant in the subject at both dates, that of the event and that of the referent time, such as sex, smoking history, occupational history, and genetics. This design was initially used to assess the effect of exposures measured at an individual level (telephone calls and traffic accidents, physical or sexual activity, and acute myocardial infarction) and was not applicable to exposures with a time trend, such as air pollution. Thus, if an investigator selected exposure control dates before the effect, and there was a trend, prior exposures would be systematically higher or lower than at the date of the effect. To circumvent this bias, Navidi (1998) developed a variant of this design, bidirectional CCO, which is conceptually characterized by having control time periods before and after the event, something that made it possible to control for the effect of long-term trend and seasonality on the variable “exposure.” This design was already appropriate for ecologic-type exposures, such as air pollution, because the existence of registries means that the values of such exposure can be ascertained even after the event. In addition, pollution values are not affected by the presence of prior morbidity and mortality events. In the CCO design, the referent time periods represent the counterfactual exposure experience of the individual, had he or she not become sick; because in air pollution pre- and postevent exposure values are independent of the hazard-period exposure, those that are postevent referent can be appropriate. One advantage of CCO design over Poisson regression is its ability to assess potential effect modification (i.e., statistical interaction) at the individual level rather than at the group level (Figueiras et al. 2005). As an alternative analytic methodology to Poisson regression, the CCO approach allows for direct modeling of interaction terms, rather than depending on multiple subgroup analyses (Figueiras et al. 2005).

We conducted a systematic review of the CCO design used to study the relationship between air pollution and morbidity and mortality, from both a methodologic and an applied standpoint.

## Materials and Methods

We conducted a bibliographic search in January 2009 using the MEDLINE (National Library of Medicine, Bethesda, MD, USA) and EMBASE (Elsevier, New York, NY, USA) databases and the key words case-crossover* and pollution*; the time frame was 1999 through 2008. From the total number of papers, we selected a series of reports based on the language used and the topic addressed in the title and/or abstract, thereby eliminating all that were not written in English or Spanish and that did not address the subject targeted for study. All the reports chosen in this way were reviewed, and additional reports were selected from among those cited in the respective references.

The reports retrieved were classified into two major groups: methodology reports in which new CCO designs were described or existing designs compared, generally by means of simulation studies, and application reports, in which some CCO design was applied for the purpose of analyzing the relationship between air pollution and health.

The methodology reports were in turn classified into those that conducted simulation studies to compare CCO designs with one another or with other designs, such as Poisson time-series, and those that described theoretical aspects pertaining to CCO design.

From the application reports, the following data were obtained for comparison: author, study location, year, dependent variable(s), independent variable(s), and type of CCO design (unidirectional, symmetric, semisymmetric, or time stratified). The modeling of interaction terms between pollutants and the individual characteristics of the subjects was also assessed, to record whether the reports had analyzed effect modification. For this purpose, only interactions with subjects’ individual variables were considered, with the following deemed ineligible: studies only reporting interactions between pollutants and pollen, meteorological variables, or other pollutants; and stratified analyses in which different models were constructed for each subgroup and no interaction term was included in a single model.

## Results

**Figure 1:**
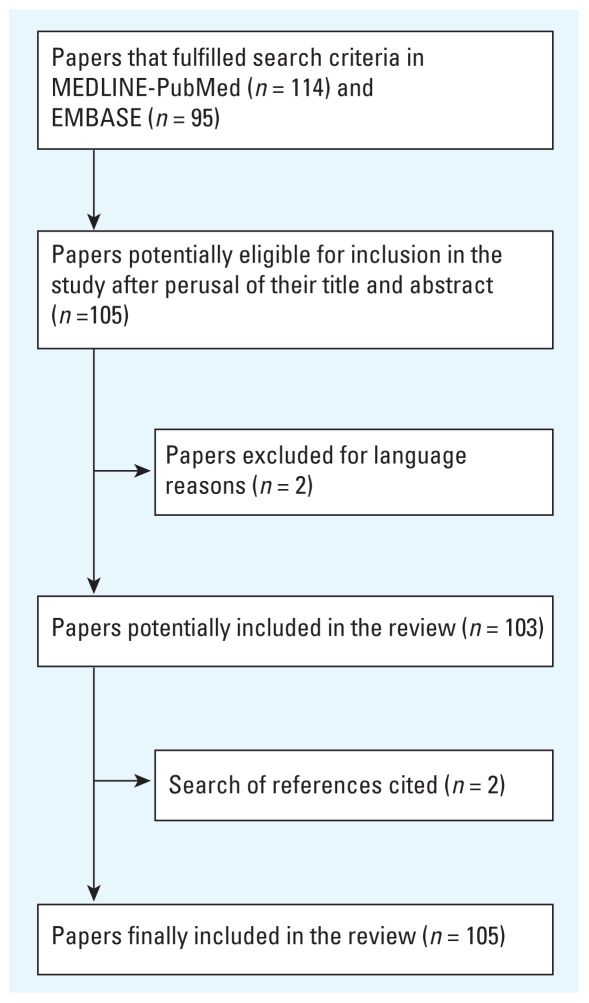
Identification of studies and inclusion criteria.

Figure 1 schematically depicts the results obtained in the bibliographic search. Of the total of 105 reports retrieved as a result of the bibliographic search, 24 addressed methodological aspects of CCO design (Bateson and Schwartz 1999, 2001; Figueiras et al. 2005; Fung et al. 2003; Hajat 2003; Jaakkola 2003; Janes et al. 2005a, 2005b; Kunzli and Schindler 2005a, 2005b; Lee et al. 2000; Levy et al. 2001a; Lu et al. 2008; Lu and Zeger 2007; Lumley and Levy 2000; Maclure 1991; Maclure and Mittleman 2008; Marshall and Jackson 1993; Mittleman 2005, Navidi 1998; Navidi et al. 1999; Navidi and Weinhandl 2002; Peters et al. 2006; Sheppard et al. 2001); the remaining studies applied CCO designs to study the relationship between different air pollutants and different outcome variables in terms of human health (Barnett et al. 2005, 2006; Bateson and Schwartz 2004; Boutin-Forzano et al. 2004; Carracedo-Martinez et al. 2008; Chang et al. 2005; Checkoway et al. 2000; Cheng et al. 2007; D’ippoliti et al. 2003; Filleul et al. 2004; Forastiere et al. 2005, 2007; Henrotin et al. 2007; Hinwood et al. 2006; Jalaludin et al. 2008; Johnston et al. 2007; Kan and Chen 2003; Karr et al. 2006; Kim et al. 2007; Kwon et al. 2001; Laurent et al. 2008; Lee and Schwartz 1999; Lee et al. 2007a, 2007b, 2008; Levy et al. 2001b; Lin et al. 2002, 2003, 2005; Ljungman et al. 2008; Luginaah et al. 2005; Maynard et al. 2007; Medina-Ramón et al. 2006; Neas et al. 1999; Peel et al. 2007; Perez et al. 2008; Peters et al. 2001, 2005; Pope et al. 2006, 2008; Rich et al. 2004, 2005, 2006a, 2006b; Romieu et al. 2004; Ruidavets et al. 2005; Schwartz 2004a, 2004b, 2005; Ségala et al. 2008; Son et al. 2008; Stafoggia et al. 2008; Sullivan et al. 2003, 2005; Sunyer and Basagana 2001; Sunyer et al. 2000, 2002; Symons et al. 2006; Tecer et al. 2008; Tsai et al. 2003a, 2003b, 2006a, 2006b; Villeneuve et al. 2006, 2007; Wellenius et al. 2005a, 2005b, 2006; Xu et al. 2008; Yamakazi et al. 2007; Yang 2008; Yang and Chen 2007; Yang et al. 2003, 2004a, 2004b, 2006b, 2007; Zanobetti and Schwartz 2005, 2006; Zeka et al. 2005, 2006).

### CCO design

Of the 24 reports that addressed CCO design, nine conducted simulation studies, one study compared the estimators obtained by different methods applied to real data, and the remaining 14 analyzed only theoretical aspects of CCO design, without performing simulations or comparisons.

Our review of methodological aspects revealed a trend in CCO bidirectional designs with regard to the choice of control periods (Table 1). The main bidirectional CCO designs, in chronological order of appearance, were as follows: *a*) full-stratum CCO, one of the designs initially proposed by Navidi (1998), in which all the days of the series except that of the event were taken as controls; *b*) random matched-pair CCO, which was also proposed by Navidi (1998) and consisted of taking any day of the series before or after the event, at random; *c*) symmetric CCO, proposed by Bateson and Schwartz (1999), which consisted of taking 2 days of the series as the controls, one before and one after the event, equidistant from the latter; *d*) time-stratified CCO, a design proposed by Lumley and Levy (2000), consisting of taking as control one or more days falling within the same time stratum as that in which the event occurred; for example, if “month” is established as the time stratum and the event occurs on, say, a Monday, then this is compared with all the Mondays in that same month; and *e*) semisymmetric CCO, proposed by Navidi and Weinhandl (2002), which consists of randomly choosing as control only one of the two controls used by symmetric CCO.

Simulation studies compare model predictions based on repeated samples drawn from a data set that represents the entire population of interest and for which true values are known because they were determined by the investigator when the data set was created in order to represent a scenario of interest. They compare the performance of different CCO designs (process or manner of functioning or operating) based on such indicators as efficiency (with relative increases in variance or standard error indicating less efficiency), bias (the difference between the model-estimated value and the true value of the parameter being estimated), and coverage (the proportion of replicate estimates that include the true value of the coefficient within their 95% confidence intervals). Simulation studies yielded the following results, in chronologic order (summarized in Table 2).

Navidi (1998), in a simulation scenario based on real data for particulate matter (PM) with aerodynamic diameter ≤ 10 μm (PM_10_) and an unmeasured confounding variable that generated a long-term trend, conducted a simulation in which unidirectional was compared with bidirectional full-stratum CCO design and observed that the bidirectional design resulted in less bias.

Bateson and Schwartz (1999), in a simulation scenario based on real PM_10_ data and an unmeasured confounding variable that generated long-term trend and seasonality (short-term trends), conducted a simulation to compare Poisson time-series regression design against different CCO designs, such as unidirectional, full-stratum, random matched pair, and symmetric, with control periods ranging from 1–4 weeks before and after the event. The results of this simulation showed that, whereas the symmetric CCO design performed best in terms of bias, it nevertheless displayed a lower efficiency (66%) than did the Poisson time-series designs.

Lumley and Levy (2000) compared symmetric with time-stratified CCO designs in a simulation scenario based on real black smoke data and an unmeasured confounding variable that generated long-term trend and seasonality; they observed better performance with the time-stratified CCO design, although both displayed a small degree of bias.

Lee et al. (2000), in a simulation scenario based on real mortality data and an unmeasured confounding variable that generated seasonality, compared unidirectional design with symmetric CCO and found that the latter performed better, although bias increased when the number of seasonality waves was incomplete.

Bateson and Schwartz (2001) set out to study the best distance at which to use control days in symmetric CCO design, in a scenario with trend and seasonality, in which all the variables were simulated. They studied control days ranging from 1–28 days before and after the event and observed that confounding was minimized when the spacing was equal to the period of exposure.

Levy et al. (2001a), in a simulation scenario based on real black smoke data and an unmeasured confounding variable that generated long-term trend but no seasonality, compared unidirectional with symmetric design, using different numbers of control periods and at different intervals from the event period, as well as the influence of autocorrelation (correlation of a temporal series variable with its own previous or posterior values) between control periods and overlapping (bias resulting from the use of incorrect referent periods), and concluded that the symmetric CCO design performed better, with less bias when the distance of the control periods from the event was 7 days and when autocorrelation and overlapping were avoided.

Navidi and Weinhandl (2002) conducted a simulation in a scenario based on real PM_10_ data and an unmeasured confounding variable that generated long-term trend and seasonality, in which they compared Poisson time-series design with the following CCO designs: symmetric with control periods separated by 7 days with respect to the case date, semisymmetric with the control period separated by 7 days with respect to the case date, random matched pair, and full-stratum. They concluded that the semisymmetric design performed best.

Fung et al. (2003) conducted a simulation in a simulation scenario based on real PM with aerodynamic diameter ≤ 2.5 μm (PM_2.5_) data and an unmeasured confounding variable that generated long-term trend and seasonality, in which they compared Poisson time-series design against unidirectional, symmetric, and semisymmetric CCO designs. They concluded that, although the symmetric design displayed a better performance in terms of bias than did the other designs studied, it was nonetheless similar to that of the Poisson time-series design, which showed a better coverage and statistical power thanks to its greater efficiency.

Figueiras et al. (2005), in a simulation study that used a simulation scenario based on real PM_10_ data and an unmeasured confounding variable that could generate long-term trend and seasonality, compared the Poisson time-series design with a number of CCO designs: symmetric, semisymmetric, time stratified, full symmetric (14 control periods before and after event) analyzed by longitudinal designs, and full semisymmetric (seven control periods before and after event) analyzed by longitudinal designs. They reported that the full semisymmetric design displayed the least bias together with the best coverage and statistical power but proved unstable when the beta value (strength of association between the pollutant and the event) varied with respect to the usual values. Although semisymmetric CCO displayed fewer biases than did symmetric or time-stratified CCO (both of which yielded similar results), it suffered from the drawback of having a lower statistical power.

It is particularly interesting to note that three of these simulation studies (Bateson and Schwartz 1999; Figueiras et al. 2005; Navidi and Weinhandl 2002) generated data for simulations using the same equations to determine trend and seasonality, before going on to use different real pollution data, such that comparable scenarios were investigated by each set of investigators.

In a separate study, Peters et al. (2006) analyzed a real database by means of a CCO and an alternative design (Poisson time-series design or Cox regression analysis) and then compared the results, observing that the time-stratified CCO design yielded results and conclusions similar to those of the Poisson time-series design and Cox regression analysis.

### CCO studies of the relationship between pollution and health

**Figure 2:**
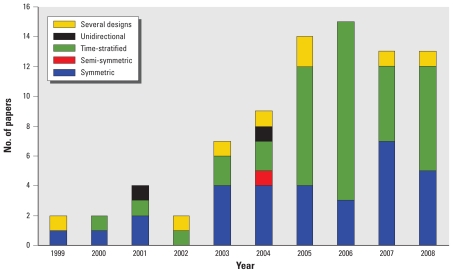
Trend in the use of different CCO methods for analyzing the short-term relationship between air pollution and health.

CCO designs are increasingly being applied to the task of analyzing the relationship between air pollution and its short-term effects on health (Figure 2). Tables 3–5 provide a detailed description of the studies published to date.

The reports published by Lee and Schwartz (1999) and Neas et al. (1999) were the first studies to report the relationship between air pollution and mortality using a CCO design. These studies performed a reanalysis of the effects of air pollution and mortality in the cities of Philadelphia and Seoul, respectively, and obtained a relationship that proved statistically significant. These results are similar to those previously obtained with the Poisson time-series design and thus strengthen the relationship of causality, inasmuch as the same relationship was observed when different statistical methods were applied.

Analysis of which CCO designs were most commonly used in the published reports showed that 7.7% of these were unidirectional and the remainder bidirectional. The most frequently used bidirectional designs were symmetric (42.2% of studies) and time stratified (48.9% of studies). The semisymmetric bidirectional design was used in only one study. Figure 2 depicts the time trend in the use of the different CCO designs. Although unidirectional designs were used in the initial period, they were gradually discarded. Most of the published studies used a 1-day control period, but six studies used a 1-hr control period.

Most of the studies that employed symmetric CCO designs used day 7 before and after the event as the control days (*n* = 23), although a variety of other schemes were also used (Table 3). Studies that used time-stratified CCO typically selected a control day on the same day of the week during the same month as the event, although other schemes (e.g., selecting days during the same month with comparable temperature) were also used (Table 4). Studies that used unidirectional CCO designs used a variety of schemes to select control days (e.g., day 7 before the event) (Table 5).

The dependent variables studied were mortality related in 25 cases and morbidity related in the remainder: hospital admissions in 35 studies, hospital emergencies in 7 studies, episodes of arrhythmias recorded in pacemakers in 5 studies, telephone calls to medical emergencies in 2 studies, and others based on disease-specific registers, such as stroke (1 study), cardiac arrest (3 studies), and ischemic heart disease (2 studies).

In 77 studies, the air pollutant analyzed was particulate level, mostly measured as PM_10_ (61 studies), followed by PM_2.5_ (22 studies), black smoke (11 studies), haze coefficient (3 studies), total suspended PM (4 studies), sulfate particles (1 study), and PM with aerodynamic diameter < 7mm (1 study). Insofar as gaseous air pollutants were concerned, sulfur dioxide was used on 47 studies, nitrogen dioxide on 48, ozone on 44, carbon monoxide on 43, and oxides of oxygen (O_x_), oxides of nitrogen (NO_x_), and nitrogen oxide on 1 study each.

In most cases, the general population was studied. Patients were studied in only 9 studies: cardiac pacemaker carriers in 5, chronic obstructive pulmonary disease patients in 2, and asthma and heart failure patients in 1 study each.

Of all the studies that addressed application of CCO designs, 11 (13.6%) made use of analysis of effect modification of variables at the individual level.

### Common steps and requirements for CCO study designs

The procedures followed in conducting a study into the relationship between air pollution and health, taking all reports on CCO design methodology and application into account, are outlined in the Appendix.

In brief, CCO studies begin by confirming that data meet a series of necessary requisites and end with a sensitivity analysis, after passing through a series of intermediate steps that include the transformation of the database into a matrix with CCO structure.

## Discussion

This is the first systematic review to cover the application of CCO designs to the study of the health effects of air pollution. Use of CCO designs has risen steeply in recent years and from 2003 in particular, reaching a peak in 2006. Most of the new CCO designs that gradually appeared were based on simulation studies, which in many cases neither relied on the same scenarios nor assessed performance for variables with special characteristics, for example, discontinuous exposures. Most application studies have tended to study the effect of particulates on morbidity, yet few studies have taken advantage of the strength of CCO designs to assess potential effect modifications with individual variables.

### CCO versus Poisson

The increase in the use of the CCO design appears to coincide with problems using Poisson regression models with GAM: as far back as 2002, Dominici et al. (2002) discovered that the most frequently used statistical packages gave rise to unstable estimators due to inadequate convergence criteria that could underestimate standard errors because of the presence of concurvity in the data (Ramsay et al. 2003). In part, the CCO design represents a solution to the problems posed by GAM methods, but before it can become generalized, a period of time is required. For instance, we observed no marked increase in the use of these designs until some years after the discovery of GAM-related problems; a peak in use occurred 2 years after the discovery of the problems of concurvity (analog to collinearity for nonlinear relationships). Currently, other (e.g., geographic) methods are also being used to analyze the link between air pollution and health (Zeger et al. 2008).

### Different CCO designs and their evolution

We observed an ongoing effort to perfect the CCO design dating from the initial unidirectional design up to the bidirectional designs with their subtypes. Successive simulation studies have focused on studying the designs that yielded the best results in previous simulations. Symmetric bidirectional CCO and time-stratified CCO most often proved to be best in different simulations. In contrast, the semisymmetric design yielded contradictory results: in some simulation studies it proved better than the symmetric design, but other studies gave opposite results (Fung et al. 2003), which could be due to differences in the simulation scenario. One consistent finding, however, is that the statistical efficiency of semisymmetric CCO is low compared with that of the symmetric or time-stratified CCO methods.

The rapid adoption of symmetric and time-stratified CCO designs is noteworthy, in that these began to be applied in the very same year in which their methodology was first proposed in the scientific literature. In contrast, the semisymmetric CCO design was first proposed in 2002, yet the first report in which it was used to analyze the relationship between air pollution and health was published in 2004.

One possible explanation for the fact that different designs are used in practice is that they were discovered at different points in time: unidirectional were described before bidirectional methods, and within bidirectional methods, symmetric was described before time-stratified CCO. Unidirectional methods are being used less frequently because of important disadvantages, such as poor control of trends.

Of the three bidirectional methods, semisymmetric is used very little because of its negligible statistical power. Symmetric and time-stratified designs had a similar percentage of use, with a trend toward greater use of time-stratified designs, possibly because, from a theoretical point of view, they solve the “overlap bias” that symmetric designs otherwise display. However, simulation studies are not conclusive when it comes to comparing time-stratified with symmetric designs; for example, in their simulation study, Lumley and Levy (2000) reported that the time-stratified method was superior, but Figueiras et al. (2005) did not find this method to be better than the symmetric CCO.

The fact that the CCO designs most often used to analyze the relationship between air pollution and health are symmetric and time stratified, plus the rapid adoption of these same two models (they began to be used in the same year as they were proposed in the literature), together indicate that there is an interest in the correct application of this methodology. Control periods most frequently used for the symmetric design are 7 days before and after case, and for the time-stratified design, control periods are all the same days of the week as the case within the same month. Thus, these two approaches prevent problems of autocorrelation, and control for effect of day of the week.

### Interpretation of application studies

In studies that use the CCO design to analyze the relationship between air pollution and health, the most frequently used exposure is that of hospital admissions. The greater use of hospital admissions than mortality as an outcome may be because, on the one hand, the hospital admission variable entails a greater number of events, thereby affording greater statistical power, and on the other hand, the time period from exposure until the event is shorter for hospital admissions than for mortality, thereby requiring a smaller number of lags, thus facilitating statistical analysis (American Thoracic Society 1985). The type of pollutant most frequently analyzed with CCO designs is airborne particulates, possibly because these have been widely studied and because exposure data are readily available. In terms of type of population, these studies seldom target diseased populations but focus instead on general populations, possibly because of the difficulty of obtaining records for a specific disease population (Filleul et al. 2004).

### Lessons learned and new challenges

Although the application of nonparametric Poisson models amounted to a great advance over earlier designs, enabling more flexible control of unmeasured confounding variables that change over time, the problems detected, such as the difficulty in setting the number of degrees of freedom, seem to have heightened interest in other alternatives, such as CCO. These approaches make it possible to control for the influence of trend and seasonality by design. Initially, these designs resulted in certain biases in the estimators under very specific conditions, which were superseded by new control period sampling designs, although a decision must still be made as to precisely what is the most appropriate time interval between case and control periods.

In principle, CCO designs seem easier to model and involve fewer arbitrary decisions for the researcher than do Poisson time-series designs with GAM (type of smoother, number of degrees of freedom), yet CCO designs also entail arbitrariness in the selection of reference periods or sampling method.

There are no known study characteristics that would favor using one referent period over another, because the heterogeneity of the simulation studies in terms of their scenarios and results renders it impossible to draw any conclusion in this regard. Likewise, simulation studies have tended to concentrate on PM, and no simulation study assesses the latter’s behavior in discontinuous exposures (e.g., a high-ozone day). In this type of exposure where high proportions of cases and controls assume a value of zero, Poisson time series might, from a theoretical point of view, perform better than CCO methods, because the comparisons are made in the same person and, when the case and control periods have the same value, provide no statistical power when analyzed with conditional logistic regression. However, we are not aware of any simulation studies that have tested whether this assumption has any relevance in practice.

Theoretically, one of the great advantages of CCO designs is that individual data can be included to estimate effect modifications, but in practice most CCO-based studies on the relationship between air pollution and health do not analyze effect modification at the individual level. The scant use of this advantage might be due to the lack of availability of data at this level (Filleul et al. 2004).

Furthermore, thanks to the CCO design, we have more scientific evidence of the short-term association between air pollution and health, because at times reanalyses using CCO methodology have been run on data previously analyzed with Poisson methods, and similar results have been obtained (Lee and Schwartz 1999).

One possible challenge is the application of mixed models to the analysis of CCO designs, something that, on the one hand, could furnish greater statistical power and, on the other, could extend CCO designs to spatial-temporal models. Figueiras et al. (2005) attempted to apply longitudinal models to CCO designs but observed that, in the presence of autocorrelation, estimates might be biased. New approaches in this field could solve these problems.

From the standpoint of statistical analysis, Lu et al. (2008) have proposed that CCO models should be checked to see if assumptions for using CCO methodology were satisfied, via a series of diagnostic tools such as plotting the data. In practice, however, we have detected no CCO study on the relationship between air pollution and health that checked the models. Furthermore there are no formulas for calculating sample size (or statistical power) in CCO designs, and indeed, one study (Symons et al. 2006) applied a simulation to calculate the lower bound of detectable effects. A possible risk of CCO designs lies in “model shopping,” whereby multiple analyses are performed using different designs, and only the most interesting are then shown (Mittleman 2005). This problem can be solved, in part, by means of a sensitivity analysis, in which the authors show the results obtained with different CCO methods, and even compare the results against a generalized linear model with a Poisson response.

### Limitations of our review

In assessing the reports that use effect modification with individual data, we encountered difficulties regarding use of different terminologies: some used the term “modification” to classify what is in reality “stratification into subgroups”; others referred to stratification but did not clarify whether different statistical models were used for each group of subjects of the variable “stratification,” or whether an interaction term was introduced into the model to assess effect modification. Furthermore, as with any systematic review, publication bias may be present.

## Conclusions

The CCO design could be an attractive alternative to Poisson time-series analysis with GAM, but its advantages and drawbacks are still in the process of being understood. The use of CCO designs to study the relationship between air pollution and health has experienced a great upsurge, but with few exceptions, full advantage has not been taken in terms of effect modification or spatial-temporal analyses. Moreover, although a number of simulations have been conducted to study the performance of CCO designs, the performance of discontinuous exposures, such as ozone, remains to be studied. A further, very important challenge would be to undertake an in-depth longitudinal analysis of CCO designs, which would enhance their statistical power and enable them to be applied to spatial-temporal models.

## Figures and Tables

**Table 1:** Comparison of different CCO designs.

Reference	Type	Selection of controls	Advantages	Factors that can introduce bias	Selection of controls diagram
Maclure 1991	CCO	One control point before theeffect	All possible confoundingfactors undergoing no changebetween control periods andeffect, automatically controlledfor by design	Long-term trends orseasonality	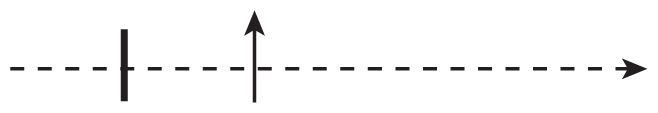
Navidi 1998	Full-stratumbidirectional	For each case, all the days ofthe series other than that ofthe event taken as controls	Provides control for long-termtrends	Long-term trends (onlypartially controlledfor) or seasonality	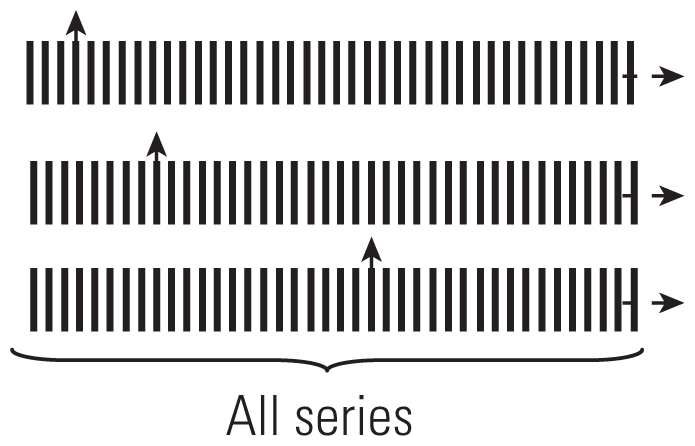
Bateson andSchwartz 1999	Symmetricbidirectional	Two at equal distance ofthe event	Provides adequate controlfor long-term trends andseasonality		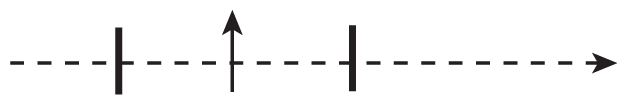
Navidi andWeinhandl 2002	Semisymmetricbidirectional	One chosen at random fromthe two used for symmetricbidirectional CCO	Provides adequate controlfor long-term trends andseasonality		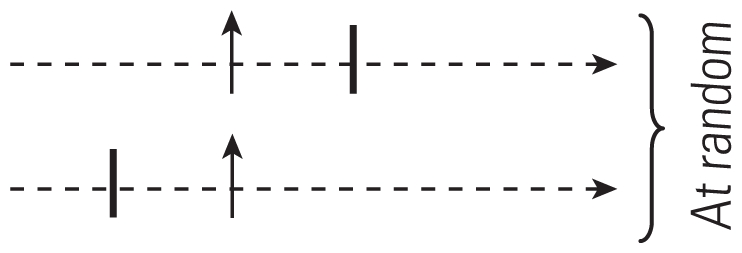
Lumley and Levy2000	Time stratified	One (or several) within thesame time stratum in whichthe event occurred	Provides adequate controlfor long-term trends andseasonality		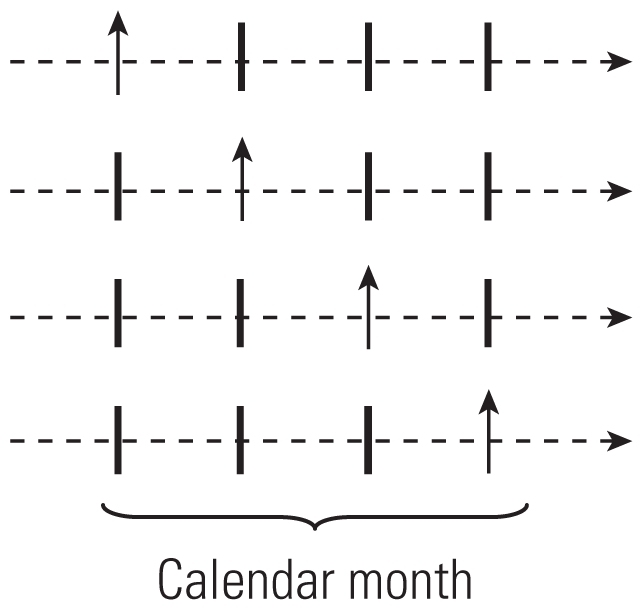

Arrows pointing up indicate case periods; horizontal arrows represent direction of time within 1 month; dashed lines indicate time periods of 1 day; vertical lines indicate control periods.

**Table 2:** Characteristics of the scenarios of simulation studies on CCO designs applied to the relationship between air pollution and health.

Reference	Long-term trend	Short-term trend (seasonality)	Pollutant^a^	Event variable^b^	Site of real data collection	Study period
Navidi 1998	Yes	No	PM_10_	S	10 communities inSouthern California	1 January 1994 to30 December 1994
Bateson and Schwartz 1999	Yes^c^	Yes^c^	PM_10_	S	Seattle	1988–1990
Lumley and Levy 2000	Yes	Yes	BS	S	King County(Washington)	1989–1994
Lee et al. 2000	No	Yes	S	Mortality	Seoul	1 October 1991 to30 September 1993
Bateson and Schwartz 2001	Yes	Yes	S	C	[Table-fn tfn2-ehp.0901485]—	3 years
Levy et al. 2001a	Yes	No	BS	S	King County(Washington)	3 October 1988 to25 June 1994
Navidi and Weinhandl 2002	Yes^c^	Yes^c^	PM_10_	S	Denver	1989–1992
Fung et al. 2003	Yes	Yes	PM_2.5_	S	Toronto	1981–1993
Figueiras et al. 2005	Yes^c^	Yes^c^	PM_10_	S	Barcelona	1995–1997

—, simulation site only.

a BS, black smoke; PM_2.5_, PM with aerodynamic diameter ≤ 2.5 μm; PM_10_, PM with aerodynamic diameter ≤ 10 μm; S, simulated.

b S, simulated (variable generated mathematically on the basis of other variables that enter into the simulation); C, created (variable generated artificially, although not on the basis of other variables that enter into the simulation).

c The simulations by Bateson and Schwartz (1999), Navidi and Weinhandl (2002), and Figueiras et al. (2005) share the same simulation scenario, in the sense that these authors use the same equation to generate trend and seasonality in the data series.

**Table 3:** Studies of air pollution health effects using symmetric CCO.

Reference	Country^a^	Study population^b^	Control period^c^	Exposure^d^	Outcome variable^e^
Neas et al. 1999	US	GP	Days (± 7, 14, 21)	TSP	Nonaccidental M
Sunyer et al. 2000	Sp	P with COPD > 35 years of age	Days (± 7)	BS	Nonaccidental M
Sunyer and Basagana 2001	Sp	P with COPD > 35 years of age	Days (± 7)	PM_10_, CO, NO_2_, O_3_	Nonaccidental M
Kwon et al. 2001	SK	P with heart failure	Days (± 7, 14)	PM_10_, CO, NO_2_, SO_2_, O_3_	Nonaccidental M
Yang et al. 2003	Ca	GP < 14 and > 65 years of age	Days (± 7)	COH, CO, NO_2_, SO_2_, O_3_	HA due to respiratory disease
Tsai et al. 2003a	Chi	GP	Days (± 7)	PM_10_, BS, CO, NO_2_, SO_2_, O_3_	HA due to stroke
Tsai et al. 2003b	Chi	GP	Days (± 7)	PM_10_, BS, CO, NO_2_, SO_2_, O_3_	Nonaccidental M
Lin et al. 2003	Ca	GP > 6 and < 12 years of age	Days (± 14)	CO, NO_2_, SO_2_, O_3_	HA due to asthma
Yang et al. 2004a	Chi	GP	Days (± 7)	PM_10_, CO, NO_2_, SO_2_, O_3_	Nonaccidental M
Yang et al. 2004b	Chi	GP	Days (± 7)	PM_10_, CO, NO_2_, SO_2_, O_3_	HA due to cardiovascular cause
Bateson and Schwartz 2004	US	GP > 65 years of age	Days (± 6–14)	PM_10_	HA due to cardiac or respiratory cause
Rich et al. 2004	Ca	P with pacemaker	Days (± 7)	PM_10_, SO_2_, NO_2_, O_3_	Cardiac arrhythmias
Chang et al. 2005	Chi	GP	Days (± 7)	PM_10_, NO_2_, CO, O_3_	HA due to cardiovascular cause
Luginaah et al. 2005	Ca	GP	Days (± 14)	PM_10_, COH, NO_2_, SO_2_, CO, O_3_	HA due to respiratory cause
Barnett et al. 2005	Au, NZ	GP < 14 years of age	Days (± 2–14)	PM_10_, PM_2.5_, COH, NO_2_, SO_2_	HA due to respiratory cause
Lin et al. 2005	Ca	GP < 14 years of age	Days (± 14)	PM_10_, PM_2.5_, SO_2_, CO, NO_2_, O_3_	HA due to respiratory infection
Yang et al. 2006	Chi	GP	Days (± 7)	PM_10_, SO_2_, CO, O_3_, NO_2_	Postneonatal M
Tsai et al. 2006b	Chi	GP	Days (± 7)	PM_10_, SO_2_, CO, O_3_, NO_2_	HA due to asthma
Tsai et al. 2006a	Chi	GP	Days (± 7)	PM_10_, SO_2_, CO, O_3_, NO_2_	Postneonatal M
Cheng et al. 2007	Chi	GP	Days (± 7)	PM_10_, SO_2_, CO, O_3_, NO_2_	HA due to pneumonia
Lee et al. 2007b	Chi	GP	Days (± 7)	PM_10_, SO_2_, CO, O_3_, NO_2_	HA due to heart failure
Yang and Chen 2007	Chi	GP	Days (± 7)	PM_10_, SO_2_, CO, O_3_, NO_2_	HA due to COPD
Yang et al. 2007	Chi	GP	Days (± 7)	PM_10_, SO_2_, CO, O_3_, NO_2_	HA due to asthma
Lee et al. 2007a	Chi	GP	Days (± 7)	PM_10_, SO_2_, CO, O_3_, NO_2_	HA due to COPD
Kim et al. 2007	SK	GP	Days (± 7), days (± 7, 14)	PM_10_, SO_2_, CO, O_3_, NO_2_	HE due to asthma
Henrotin et al. 2007	Fr	GP	Days (± 7, 14, 21, 28)	PM_10_, SO_2_, CO, O_3_, NO_x_	Stroke
Ségala et al. 2008	Fr	GP < 3 years of age	Days (± 7–8, 14–15)	PM_10_, BS, SO_2_, NO_2_	HE due to bronchiolitis
Yang 2008	Chi	GP	Days (± 7)	PM_10_, SO_2_, NO_2_, CO, O_3_	HA heart failure
Tecer et al. 2008	Tu	GP < 14 years of age	Days (± 7–14)	PM_10_, PM_2.5_	HA respiratory diseases
Carracedo-Martinez et al.2008	Sp	GP	Days (± 7)	BS, SO_2_	ETC due to respiratory andcardiovascular causes
Son et al. 2008	SK	GP	Days (± 7), days (± 7, 14), days (± 7, 14, 21)	PM_10_, SO_2_, NO_2_, CO, O_3_	Postneonatal M

a Au, Australia; Ca, Canada; Chi, China; Fr, France; NZ, New Zealand; SK, South Korea; Sp, Spain; Tu, Turkey; US, United States of America.

b COPD, chronic obstructive pulmonary disease; GP, general population; P, patients.

c Interpretation of control periods: days (±7), 7th day before and after the case; days (±7, 14), days 7 and 14 before and after the case; days (± 7–14), days 7–14 before and after the case.

d BS, black smoke; CO, carbon monoxide; COH, PM measured as haze coefficient; NO_2_, nitrogen dioxide; NO_x_, nitrogen oxide; O_3_, ozone; PM_10_, PM with aerodynamic diameter ≤ 10 μm; PM_2.5_, PM with aerodynamic diameter ≤ 2.5 μm; SO_2_, sulfur dioxide; TSP, total suspended PM.

e ETC, emergency telephone calls; HA, hospital admission; HE, hospital emergency; M, mortality.

**Table 4:** Studies of air pollution health effects using time-stratified CCO.

Reference	Country^a^	Study population^b^	Control period^c^	Exposure^d^	Outcome variable^e^
Checkoway et al. 2000	US	GP	=month, =weekday	BS, PM_10_, SO_2_, CO	Cardiac arrest
Levy et al. 2001b	US	GP	=month, =weekday	PM_2.5_, PM_10_	Cardiac arrest
Sunyer et al. 2002	Sp	Asthmatic P > 14 yearsof age	=month, =weekday	PM_10_, BS, CO, NO_2_, SO_2_, O_3_	M due to asthma
Sullivan et al. 2003	US	GP	=month, =weekday	PM_10_, CO, SO_2_	Cardiac arrest
D’Ippoliti et al. 2003	Ita	GP	=month, =weekday	TSP, CO, NO_2_, SO_2_	HA due to myocardial infarction
Schwartz 2004b	US	GP	=month, =weekday	PM_10_	M accidental
Romieu et al. 2004	Mex	GP >1 month and1 year of age	=month, =weekday	PM_10_	M due to respiratory cause
Schwartz 2005	US	GP	=month, days =temperature	O_3_	Nonaccidental M
Sullivan et al. 2005	US	GP	=month, =weekday	PM_10_, PM_2.5_, SO_2_, CO	Myocardial infarction
Wellenius et al. 2005a	US	GP > 65 years of age	=month, =weekday	PM_10_, CO, NO_2_, SO_2_, O_3_	HA due to heart failure
Rich et al. 2005	US	P with pacemaker	=month, =weekday, =hour	PM_2.5_	Cardiac arrhythmias
Forastiere et al. 2005	Ita	GP	=month, =weekday	PM_10_, CO, NO_2_, O_3_	Out-of-hospital cardiovascular M
Zanobetti and Schwartz2005	US	GP	=month, =weekday	PM_10_	HA due to myocardial infarction
Zeka et al. 2005	US	GP	=month, =weekday	PM_10_	Nonaccidental M
Wellenius et al. 2005b	US	GP	=month, =weekday	PM_10_, SO_2_, CO, NO_2_	HA due to stroke
Pope et al. 2006	US	GP	=month, =weekday	PM_2.5_, PM_10_	Ischemic coronary events
Villeneuve et al. 2006	Ca	GP > 65 years of age	=month, =weekday	PM_10_, PM_2.5_, SO_2_, CO, O_3_,NO_2_	HE due to ischemic stroke
Symons et al. 2006	US	GP	=month, =weekday	PM_2.5_	HA due to heart failure
Zeka et al. 2006	US	GP	=month, =weekday	PM_10_	Nonaccidental, cardiovascularand respiratory M
Medina-Ramon et al. 2006	US	GP	=month, =weekday	PM_10_, O_3_	HA due to pneumonia, COPD
Rich et al. 2006b	US	P with pacemaker	=month, =weekday, =hour	BS, PM_2.5_, SO_2_, CO, O_3_, NO_2_	Paroxysmal auricular fibrillation Ep
Wellenius et al. 2006	US	GP > 65 years of age	=month, =weekday	PM_10_	HA heart failure
Karr et al. 2006	US	GP < 1 year of age	=month, =weekday	PM_2.5_, CO, NO_2_	HA due to bronchiolitis
Rich et al. 2006a	US	P with pacemaker	=month, =weekday, =hour	PM_2.5_, SO_2_, CO, O_3_, NO_2_	Ventricular arrhythmia Ep
Zanobetti and Schwartz2006	US	GP > 65 years of age	=month, days =temperature	PM_2.5_, BS, CO, O_3_, NO_2_	HA due to myocardial infarction and pneumonia
Hinwood et al. 2006	Au	GP	=month, =weekday	BS, PM_10_, PM_2.5_, CO, O_3_,NO_2_	HA due to cardiovascular andrespiratory disease
Barnett et al. 2006	Au, NZ	GP > 15 years of age	=month, all days but day ±1	PM_10_, PM_2.5_, SO_2_, CO, O_3_, NO_2_	HA due to cardiovascular causes
Forastiere et al. 2007	Ita	GP	=month, =weekday	PM_10_	Nonaccidental M
Peel et al. 2007	Ca	GP	=month, =weekday	PM_10_, SO_2_, CO, O_3_, NO_2_	HE due to cardiovascular causes.
Johnston et al. 2007	Au	GP	=month, =weekday	PM_10_	HA due to cardiovascular and respiratory causes
Maynard et al. 2007	US	GP	=month, all days but 2 days between	BS, sulfate particles	Nonaccidental, cardiovascular and respiratory M
Yamakazi et al. 2007	Jap	GP > 65 years of age	=month, =weekday, =hour	PM_7_, NO_2_, Ox	M due to stroke
Jalaludin et al. 2008	Au	GP > 1 and < 14 years of age	=month, all days	PM_10_, PM_2.5_, SO_2_, NO_2_,O_3_, CO,	HE due to asthma
Perez et al. 2008	Sp	GP	=month, =weekday	PM_10_, PM_2.5_	Nonaccidental M
Laurent et al. 2008	Fr	GP	=month, =weekday	PM_10_ SO_2_, NO_2_, O_3_	ETC due to asthma
Lee et al. 2008	Chi	GP	=month, =weekday	PM_10_, SO_2_, NO_2_, CO, O_3_	HA heart failure
Pope et al. 2008	US	GP	=month, =weekday	PM_10_, PM_2.5_	HA heart failure
Ljungman et al. 2008	Sw	P with pacemaker	=month, =weekday, =hour	PM_10_, NO_2_	Ventricular arrhythmia Ep
Stafoggia et al. 2008	Ita	GP > 35 years of age	=month, all days but 1 day between	PM_10_	Nonaccidental M

a Au, Australia; Ca, Canada; Chi, China; Fr, France; Ita, Italy; Jap, Japan; Mex, Mexico; NZ, New Zealand; Sp, Spain; Sw, Sweden; US, United States of America.

b GP, general population; P, patients.

c Control periods: =month, =weekday, all the days of the same month as that of the case, which was the same day of the week; =month, =weekday, =hour, hours that coincide with those of the case, on days in the same month as the case, which were the same days of the week; =month, days =temperature, days in the same month as and having a temperature equal to that of the case date; =month, all days but 2 days between, all days in the same month as that of the case except 2 days between each control day.

d BS, black smoke; CO, carbon monoxide; NO_2_, nitrogen dioxide; O_3_, ozone; PM_10_, PM with aerodynamic diameter ≤ 10 μm; PM_2.5_, PM with aerodynamic diameter ≤ 2.5 μm; SO_2_, sulfur dioxide; TSP, total suspended PM.

e Ep, episode; ETC, emergency telephone calls; HA, hospital admission; HE, hospital emergency; M, mortality.

**Table 5:** Studies of air pollution health effects using multiple CCO designs or those other than symmetric or time stratified.

Reference	Country^a^	Study population^b^	Type of CCO design^c^	Exposure^d^	Outcome variable^e^
Lee and Schwartz 1999	SK	GP	U(–7d); U(–7, 14d); U(+7d); U(+7, 14d); SB(± 7d)	TSP, SO_2_, O_3_	Nonaccidental M
Peters et al. 2001	US	GP	U(–2, 3, 4d)	PM_2.5_	Myocardial infarction
Lin et al. 2002	Ca	GP 6–12 years of age	U(–14d); SB(± 14d)	PM_10_, PM_2.5_	HA due to asthma
Kan and Chen 2003	Chi	GP	U(–7, 14, 21d); SB(± 7, 14, 21d)	PM_10_, NO_2_, SO_2_	Nonaccidental M
Filleul et al. 2004	Fr	GP > 65 years of age	SSB(± 7d)	BS	Nonaccidental M and cardiovascular M
Boutin-Forzano et al. 2004	Fr	GP > 3 and < 49 years of age	U(–7d)	SO_2_, NO_2_, O_3_	HE due to asthma
Schwartz 2004a	US	GP	SB(± 7d); TS(m, d =T)	PM_10_	Nonaccidental M
Ruidavets et al. 2005	Fr	GP	U(–7, 14, 21, 28d); SB(± 7d)	O_3_, SO_2_, NO_2_	HA due to myocardial infarction
Peters et al. 2005	Ger	GP	U(–(1–3)d ); U(–(1–3)d, =h);SB(± 7, 14d); SB(± 7, 14d, =h); SB(± 7–14d); SB(±7–14d, =h); TS(m, =wd) ; TS(m, =wd, =h)	PM_10_, PM_2.5_, TSP, SO_2_, CO, NO, NO_2_, O_3_	HA due to myocardial infarction
Villeneuve et al. 2007	Ca	GP	SB(± 7, 14d); TS(m, =wd)	PM_10_, PM_2.5_, SO_2_, CO, O_3_, NO_2_	HE due to asthma
Xu et al. 2008	US	GP	SB(± 7, 14d); TS(m, =wd)	PM_10_, SO_2_	HA respiratory and cardiovascular diseases

a Ca, Canada; Chi, China; Fr, France; Ger, Germany; SK, South Korea; US, United States of America.

b GP, general population; P, patients.

c SB, symmetric bidirectional CCO; SSB, semisymmetric bidirectional CCO; TS, time-stratified CCO; U, unidirectional CCO. Interpretation of control periods: (±7d), 7th day before and after the case; (±7; 14d), days 7 and 14 before and after the case; (±7–14d), days 7–14 before and after the case; (m, =wd), all the days of the same month as that of the case, which was the same day of the week; (m; =wd; =h), hours that coincide with those of the case, on days in the same month as the case, which were the same days of the week; (m; d =T), days in the same month as and having a temperature equal to that of the case date; U(–7d), one control day, 7 days before the case.

d BS, black smoke; CO, carbon monoxide; NO_2_, nitrogen dioxide; O_3_, ozone; PM_10_, PM with aerodynamic diameter ≤ 10 μm; PM_2.5_, PM with aerodynamic diameter ≤ 2.5 μm; SO_2_, sulfur dioxide; TSP, total suspended PM.

e HA, hospital admission; HE, hospital emergency; M, mortality.

## Appendix Applying CCO Designs to Study the Relationship between Air Pollution and Health

The steps to be followed to conduct a study into the relationship between air pollution and health, taking all reports on CCO design methodology and application into account, can be summarized as follows:

1. Confirm that the study variables meet the conditions for being able to study the association using a CCO:
  - Exposure variables must be transitory (prolonged exposures such as radon would not be valid).
  - Event variables must be acute (events such as cancer would not be valid).
  - Proportion of missing data must be small.
2. The databases obtained can be classified into one of the following types:
  - Contain only ecologic temporal cluster data.
  - Contain ecologic temporal and spatial cluster data.
  - Individual data available—this enables effect modification to be subsequently studied at the level of variables having characteristics pertaining to individuals.
3. For exposure variables, compute the individual (0, 1, 2, 3) or combined lags (0, 1, 2–3 . . .) depending on the nature of the dependent variable (longer lags are needed for mortality than for morbidity variables).
4. Transform the database into a matrix with a CCO structure, that is, with as many strata as there are events, and in each stratum there is a case period that would be formed by exposure at the time of the event (or the corresponding lag) and one (or more) control periods that would be formed by exposure in the periods selected as controls (e.g., in a symmetric CCO design, these could be day 7 before and after the event). For an ecologic database consisting solely of temporal cluster data, calculations are simplified because:
  - There are macros in S-Plus that transform an ecological matrix into a symmetric CCO, semisymmetric CCO, or time-stratified CCO (these may be requested from the corresponding author).
  - There is the possibility of conducting CCO studies using an ecologic matrix, with weighting for the daily number of events in the regression models. The advantages are that transformation into a CCO matrix is not necessary, the size of the database is smaller, and computing time is shorter.
5. To relate dependent and independent variables, perform the statistical analysis according to the following steps:
  - Construct a baseline model by introducing variables, such as temperature, ambient humidity, and atmospheric pressure. For these types of environmental variables, nonlinear risk exposure relationships might have to be checked. For the purpose, use can be made of different smoothers, such as natural splines, penalized splines, or smoothing splines. To decide whether a variable is retained in the model or the number of degrees of freedom of the smooth function, use the minimization criterion of the Akaike information criterion (Figueiras and Cadarso-Suarez 2001).
  - Construct the single-pollutant models by adding the pollutants to the baseline model.
  - Construct the multipollutant models by adding those pollutants to the baseline model that have obtained a given *p*-value in the single-pollutant model.
  - Analyze possible effect modification by reference to the statistical significance of the interaction term.
  - Analyze statistical power (Symons et al. 2006).
6. Check the models according to the method proposed by Lu et al. (2008).
7. Conduct a sensitivity analysis by analyzing the models using another type of CCO design or even a Poisson time series.
8. Report the results obtained.

## References

[b1-ehp.0901485] American Thoracic Society (1985). Guidelines as to what constitutes an adverse respiratory health effect, with special reference to epidemiologic studies of air pollution. Am Rev Respir Dis.

[b2-ehp.0901485] Barnett AG, Williams GM, Schwartz J, Best TS, Neller AH, Petroeschevsky (2006). The effects of air pollution on hospitalizations for cardiovascular disease in elderly people in Australian and New Zealand cities. Environ Health Perspect.

[b3-ehp.0901485] Barnett AG, Williams GM, Schwartz J, Neller AH, Best TL, Petroeschevsky AL (2005). Air pollution and child respiratory health: a case-crossover study in Auckland New Zealand. Am J Respir Crit Care Med.

[b4-ehp.0901485] Bateson TF, Schwartz J (1999). Control for seasonal variation and time trend in case-crossover studies of acute effects of environmental exposures. Epidemiology.

[b5-ehp.0901485] Bateson TF, Schwartz J (2001). Selection bias and confounding in case-crossover analyses of environmental time-series data. Epidemiology.

[b6-ehp.0901485] Bateson TF, Schwartz J (2004). Who is sensitive to the effects of particulate air pollution on mortality? A case-crossover analysis of effect modifiers. Epidemiology.

[b7-ehp.0901485] Boutin-Forzano S, Adel N, Gratecos L, Jullian H, Garnier JM, Ramadour M (2004). Visits to the emergency room for asthma attacks and short-term variations in air pollution. A case-crossover study. Respiration.

[b8-ehp.0901485] Carracedo-Martinez E, Sanchez C, Taracido M, Saez M, Jato V, Figueiras A (2008). Effect of short-term exposure to air pollution and pollen on medical emergency calls: a case-crossover study in Spain. Allergy.

[b9-ehp.0901485] Chang CC, Tsai SS, Ho SC, Yang CY (2005). Air pollution and hospital admissions for cardiovascular disease in Taipei, Taiwan. Environ Res.

[b10-ehp.0901485] Checkoway H, Levy D, Sheppard L, Kaufman J, Koenig J, Siscovick D (2000). A case-crossover analysis of fine particulate matter air pollution and out-of-hospital sudden cardiac arrest. Res Rep Health Eff Inst.

[b11-ehp.0901485] Cheng MF, Tsai SS, Wu TN, Chen PS, Yang CY (2007). Air pollution and hospital admissions for pneumonia in a tropical city: Kaohsiung, Taiwan. J Toxicol Environ Health.

[b12-ehp.0901485] D’Ippoliti D, Forastiere F, Ancona C, Agabiti N, Fusco D, Michelozzi P (2003). Air pollution and myocardial infarction in Rome: a case-crossover analysis. Epidemiology.

[b13-ehp.0901485] Dominici F, McDermott A, Zeger SL, Samet JM (2002). On the use of generalized additive models in time-series studies of air pollution and health. Am J Epidemiol.

[b14-ehp.0901485] Figueiras A, Cadarso-Suarez C (2001). Application of nonparametric models for calculating odds ratios and their confidence intervals for continuous exposures. Am J Epidemiol.

[b15-ehp.0901485] Figueiras A, Carracedo-Martinez E, Saez M, Taracido M (2005). Analysis of case-crossover designs using longitudinal approaches: a simulation study. Epidemiology.

[b16-ehp.0901485] Filleul L, Rondeau V, Cantagrel A, Dartigues JF, Tessier JF (2004). Do subject characteristics modify the effects of particulate air pollution on daily mortality among the elderly?. J Occup Environ Med.

[b17-ehp.0901485] Firket J (1931). The cause of symptoms found in the Meuse Valley during the fog of December, 1930. Bull Acad R Med Belg.

[b18-ehp.0901485] Forastiere F, Stafoggia M, Picciotto S, Bellander T, D’Ippoliti D, Lanki T (2005). A case-crossover analysis of out-of-hospital coronary deaths and air pollution in Rome, Italy. Am J Respir Crit Care Med.

[b19-ehp.0901485] Forastiere F, Stafoggia M, Tasco C, Picciotto S, Agabiti N, Cesaroni G (2007). Socioeconomic status, particulate air pollution, and daily mortality: differential exposure or differential susceptibility. Am J Ind Med.

[b20-ehp.0901485] Fung KY, Krewski D, Chen Y, Burnett R, Cakmak S (2003). Comparison of time series and case-crossover analyses of air pollution and hospital admission data. Int J Epidemiol.

[b21-ehp.0901485] Hajat S (2003). Commentary: comparison of time series and case-crossover analyses of air pollution and hospital admission data. Int J Epidemiol.

[b22-ehp.0901485] Henrotin JB, Besancenot JP, Bejot Y, Giroud M (2007). Short-term effects of ozone air pollution on ischaemic stroke occurrence: a case-crossover analysis from a 10-year population-based study in Dijon, France. Occup Environ Med.

[b23-ehp.0901485] Hinwood AL, De Klerk N, Rodríguez C, Jacoby P, Runnion T, Rye P (2006). The relationship between changes in daily air pollution and hospitalizations in Perth, Australia 1992–1998: a case-crossover study. Int J Environ Health Res.

[b24-ehp.0901485] Jaakkola JJ (2003). Case-crossover design in air pollution epidemiology. Eur Respir J Suppl.

[b25-ehp.0901485] Jalaludin B, Khalaj B, Sheppeard V, Morgan G (2008). Air pollution and ED visits for asthma in Australian children: a case-crossover analysis. Int Arch Occup Environ Health.

[b26-ehp.0901485] Janes H, Sheppard L, Lumley T (2005a). Case-crossover analyses of air pollution exposure data: referent selection strategies and their implications for bias. Epidemiology.

[b27-ehp.0901485] Janes H, Sheppard L, Lumley T (2005b). Overlap bias in the case-crossover design, with application to air pollution exposures. Stat Med.

[b28-ehp.0901485] Johnston FH, Bailie RS, Pilotto LS, Hanigan IC (2007). Ambient biomass smoke and cardio-respiratory hospital admissions in Darwin, Australia. BMC Public Health.

[b29-ehp.0901485] Kan H, Chen B (2003). A case-crossover analysis of air pollution and daily mortality in Shanghai. J Occup Health.

[b30-ehp.0901485] Karr C, Lumley T, Shepherd K, Davis R, Larson T, Ritz B (2006). A case-crossover study of wintertime ambient air pollution and infant bronchiolitis. Environ Health Perspect.

[b31-ehp.0901485] Kelsall JE, Samet JM, Zeger SL, Xu J (1997). Air pollution and mortality in Philadelphia, 1974–1988. Am J Epidemiol.

[b32-ehp.0901485] Kim SY, O’Neill MS, Lee JT, Cho Y, Kim J, Kim H (2007). Air pollution, socioeconomic position, and emergency hospital visits for asthma in Seoul, Korea. Int Arch Occup Environ Health.

[b33-ehp.0901485] Kunzli N, Schindler C (2005a). A call for reporting the relevant exposure term in air pollution case-crossover studies. J Epidemiol Community Health.

[b34-ehp.0901485] Kunzli N, Schindler C (2005b). Case-crossover studies. Epidemiology.

[b35-ehp.0901485] Kwon HJ, Cho SH, Nyberg F, Pershagen G (2001). Effects of ambient air pollution on daily mortality in a cohort of patients with congestive heart failure. Epidemiology.

[b36-ehp.0901485] Laurent O, Pedrono G, Segala C, Filleul L, Havard S, Deguen S (2008). Air pollution, asthma attacks, and socioeconomic deprivation: a small-area case-crossover study. Am J Epidemiol.

[b37-ehp.0901485] Lee IM, Tsai SS, Chang CC, Ho CK, Yang CY (2007a). Air pollution and hospital admissions for chronic obstructive pulmonary disease in a tropical city: Kaohsiung, Taiwan. Inhal Toxicol.

[b38-ehp.0901485] Lee IM, Tsai SS, Ho CK, Chiu HF, Wu TN, Yang CY (2008). Air pollution and hospital admissions for congestive heart failure: are there potentially sensitive groups?. Environ Res.

[b39-ehp.0901485] Lee IM, Tsai SS, Ho CK, Chiu HF, Yang CY (2007b). Air pollution and hospital admissions for congestive heart faliure in a tropical city: Kaohsiung, Taiwan. Inhal Toxicol.

[b40-ehp.0901485] Lee JT, Kim H, Schwartz J (2000). Bidirectional case-crossover studies of air pollution: bias from skewed and incomplete waves. Environ Health Perspect.

[b41-ehp.0901485] Lee JT, Schwartz J (1999). Reanalysis of the effects of air pollution on daily mortality in Seoul, Korea: a case-crossover design. Environ Health Perspect.

[b42-ehp.0901485] Levy D, Lumley T, Sheppard L, Kaufman J, Checkoway H (2001a). Referent selection in case-crossover analyses of acute health effects of air pollution. Epidemiology.

[b43-ehp.0901485] Levy D, Sheppard L, Checkoway H, Kaufman J, Lumley T, Koenig J (2001b). A case-crossover analysis of particulate matter air pollution and out-of-hospital primary cardiac arrest. Epidemiology.

[b44-ehp.0901485] Lin M, Chen Y, Burnett RT, Villeneuve PJ, Krewski D (2002). The influence of ambient coarse particulate matter on asthma hospitalization in children: case-crossover and time-series analyses. Environ Health Perspect.

[b45-ehp.0901485] Lin M, Chen Y, Burnett RT, Villeneuve PJ, Krewski D (2003). Effect of short-term exposure to gaseous pollution on asthma hospitalisation in children: a bi-directional case-crossover analysis. J Epidemiol Community Health.

[b46-ehp.0901485] Lin M, Stieb DM, Chen Y (2005). Coarse particulate matter and hospitalization for respiratory infections in children younger than 15 years in Toronto: a case-crossover analysis. Pediatrics.

[b47-ehp.0901485] Ljungman PL, Berglind N, Holmgren C, Gadler F, Edvardsson N, Pershagen G (2008). Rapid effects of air pollution on ventricular arrhythmias. Eur Heart J.

[b48-ehp.0901485] Logan WPD (1953). Mortality in the London fog incident, 1952. Lancet.

[b49-ehp.0901485] Loomis D, Richardson DB, Elliott L (2005). Poisson regression analysis of ungrouped data. Occup Environ Med.

[b50-ehp.0901485] Lu Y, Symons JM, Geyh AS, Zeger SL (2008). An approach to checking case-crossover analyses based on equivalence with time-series methods. Epidemiology.

[b51-ehp.0901485] Lu Y, Zeger SL (2007). On the equivalence of case-crossover and time series methods in environmental epidemiology. Biostatistics.

[b52-ehp.0901485] Luginaah IN, Fung KY, Gorey KM, Webster G, Wills C (2005). Association of ambient air pollution with respiratory hospitalization in a government-designated “area of concern”: the case of Windsor, Ontario. Environ Health Perspect.

[b53-ehp.0901485] Lumley T, Levy D (2000). Bias in the case-crossover design: implications for studies of air pollution. Environmetrics.

[b54-ehp.0901485] Maclure M (1991). The case-crossover design: a method for studying transient effects on the risk of acute events. Am J Epidemiol.

[b55-ehp.0901485] Maclure M, Mittleman MA (2008). Case-crossover designs compared with dynamic follow-up designs. Epidemiology.

[b56-ehp.0901485] Marshall RJ, Jackson RT (1993). Analysis of case-crossover designs. Stat Med.

[b57-ehp.0901485] Maynard D, Coull BA, Gryparis A, Schwartz J (2007). Mortality risk associated with short-term exposure to traffic particles and sulfates. Environ Health Perspect.

[b58-ehp.0901485] Medina-Ramón M, Zanobetti A, Schwartz J (2006). The effect of ozone and PM10 on hospital admissions for pneumonia and chronic obstructive pulmonary disease: a national multicity study. Am J Epidemiol.

[b59-ehp.0901485] Mittleman MA (2005). Optimal referent selection strategies in case-crossover studies: a settled issue. Epidemiology.

[b60-ehp.0901485] Navidi W (1998). Bidirectional case-crossover designs for exposures with time trends. Biometrics.

[b61-ehp.0901485] Navidi W, Thomas D, Langholz B, Stram D (1999). Statistical methods for epidemiologic studies of the health effects of air pollution. Res Rep Health Eff Inst.

[b62-ehp.0901485] Navidi W, Weinhandl E (2002). Risk set sampling for case-crossover designs. Epidemiology.

[b63-ehp.0901485] Neas LM, Schwartz J, Dockery D (1999). A case-crossover analysis of air pollution and mortality in Philadelphia. Environ Health Perspect.

[b64-ehp.0901485] Peel JL, Metzger KB, Klein M, Flanders WD, Mulholland JA, Tolbert PE (2007). Ambient air pollution and cardiovascular emergency department visits in potentially sensitive groups. Am J Epidemiol.

[b65-ehp.0901485] Perez L, Tobias A, Querol X, Künzli N, Pey J, Alastuey A (2008). Coarse particles from Saharan dust and daily mortality. Epidemiology.

[b66-ehp.0901485] Peters A, Dockery DW, Muller JE, Mittleman MA (2001). Increased particulate air pollution and the triggering of myocardial infarction. Circulation.

[b67-ehp.0901485] Peters A, von Klot S, Berglind N, Hörmann A, Löwel H, Nyberg F (2006). Comparison of different methods in analyzing short-term air pollution effects in a cohort study of susceptible individuals. Epidemiol Perspect Innov.

[b68-ehp.0901485] Peters A, von Klot S, Heier M, Trentinaglia I, Cyrys J, Hörmann A (2005). Particulate air pollution and nonfatal cardiac events. Part I. Air pollution, personal activities, and onset of myocardial infarction in a case-crossover study. Res Rep Health Eff Inst.

[b69-ehp.0901485] Pope CA, Muhlestein JB, May HT, Renlund DG, Anderson JL, Horne BD (2006). Ischemic heart disease events triggered by short-term exposure to fine particulate air pollution. Circulation.

[b70-ehp.0901485] Pope CA, Renlund DG, Kfoury AG, May HT, Horne BD (2008). Relation of heart failure hospitalization to exposure to fine particulate air pollution. Am J Cardiol.

[b71-ehp.0901485] Ramsay TO, Burnett RT, Krewski D (2003). The effect of concurvity in generalized additive models linking mortality to ambient particulate matter. Epidemiology.

[b72-ehp.0901485] Rich DQ, Kim MH, Turner JR, Mittleman MA, Schwartz J, Catalana PJ (2006a). Association of ventricular arrhythmias detected by implantable cardioverter defibrillator and ambient air pollutants in the St Louis, Missouri metropolitan area. Occup Environ Med.

[b73-ehp.0901485] Rich DQ, Mittleman MA, Link MS, Schwartz J, Luttmann-Gibson H, Catalana PJ (2006b). Increased risk of paroxysmal atrial fibrillation episodes associated with acute increases in ambient air pollution. Environ Health Perspect.

[b74-ehp.0901485] Rich DQ, Schwartz J, Mittleman MA, Link M, Luttmann-Gibson H, Catalano PJ (2005). Association of short-term ambient air pollution concentrations and ventricular arrhythmias. Am J Epidemiol.

[b75-ehp.0901485] Rich KE, Petkau J, Vedal S, Brauer M (2004). A case-crossover analysis of particulate air pollution and cardiac arrhythmia in patients with implantable cardioverter defibrillators. Inhal Toxicol.

[b76-ehp.0901485] Romieu I, Ramirez-Aguilar M, Moreno-Macias H, Barraza-Villarreal A, Millar P, Hernández-Cadena L (2004). Infant mortality and air pollution: modifying effect by social class. J Occup Environ Med.

[b77-ehp.0901485] Ruidavets JB, Cournot M, Cassadou S, Giroux M, Meybeck M, Ferrieres J (2005). Ozone air pollution is associated with acute myocardial infarction. Circulation.

[b78-ehp.0901485] Saez M, Perez-Hoyos S, Tobias A, Saurina C, Barceló MA, Ballester F (1999). Métodos de series temporales en los studies epidemiológicos sobre contaminación atmosférica [in Spanish]. Rev Esp Salud Publica.

[b79-ehp.0901485] Schwartz J (2004a). Is the association of airborne particles with daily deaths confounded by gaseous air pollutants? An approach to control by matching. Environ Health Perspect.

[b80-ehp.0901485] Schwartz J (2004b). The effects of particulate air pollution on daily deaths: a multi-city case crossover analysis. Occup Environ Med.

[b81-ehp.0901485] Schwartz J (2005). How sensitive is the association between ozone and daily deaths to control for temperature?. Am J Respir Crit Care Med.

[b82-ehp.0901485] Ségala C, Poizeau D, Mesbah M, Willems S, Maidenberg M (2008). Winter air pollution and infant bronchiolitis in Paris. Environ Res.

[b83-ehp.0901485] Sheppard L, Levy D, Checkoway H (2001). Correcting for the effects of location and atmospheric conditions on air pollution exposures in a case-crossover study. J Expo Anal Environ Epidemiol.

[b84-ehp.0901485] Son JY, Cho YS, Lee JT (2008). Effects of air pollution on postneonatal infant mortality among firstborn infants in Seoul, Korea: case-crossover and time-series analyses. Arch Environ Occup Health.

[b85-ehp.0901485] Stafoggia M, Schwartz J, Forastiere F, Perucci CA, SISTI Group (2008). Does temperature modify the association between air pollution and mortality? A multicity case-crossover analysis in Italy. Am J Epidemiol.

[b86-ehp.0901485] Sullivan J, Ishikawa N, Sheppard L, Siscovick D, Checkoway H, Kaufman J (2003). Exposure to ambient fine particulate matter and primary cardiac arrest among persons with and without clinically recognized heart disease. Am J Epidemiol.

[b87-ehp.0901485] Sullivan J, Sheppard L, Schreuder A, Ishikawa N, Siscovick D, Kaufman J (2005). Relation between short-term fine-particulate matter exposure and onset of myocardial infarction. Epidemiology.

[b88-ehp.0901485] Sunyer J, Basagana X (2001). Particles, and not gases, are associated with the risk of death in patients with chronic obstructive pulmonary disease. Int J Epidemiol.

[b89-ehp.0901485] Sunyer J, Basagaña X, Belmonte J, Antó JM (2002). Effect of nitrogen dioxide and ozone on the risk of dying in patients with severe asthma. Thorax.

[b90-ehp.0901485] Sunyer J, Schwartz J, Tobias A, Macfarlane D, Garcia J, Antó JM (2000). Patients with chronic obstructive pulmonary disease are at increased risk of death associated with urban particle air pollution: a case-crossover analysis. Am J Epidemiol.

[b91-ehp.0901485] Symons JM, Wang L, Guallar E, Howell E, Dominici F, Schwab M (2006). A Case-crossover study of fine particulate matter air pollution and onset of congestive heart failure symptom exacerbation leading to hospitalization. Am J Epidemiol.

[b92-ehp.0901485] Tecer LH, Alagha O, Karaca F, Tuncel G, Eldes N (2008). Particulate matter [PM_(2.5)_, PM_(10–2.5)_, and PM_(10)_] and children’s hospital admissions for asthma and respiratory diseases: a bidirectional case-crossover study. J Toxicol Environ Health A.

[b93-ehp.0901485] Tsai SS, Chen CC, Hisieh HJ, Chang CC, Yang CY (2006a). Air pollution and postneonatal mortality in a tropical city: Kaohsiung, Taiwan. Inhal Toxicol.

[b94-ehp.0901485] Tsai SS, Cheng M, Chiu H, Wu T, Yang CY (2006b). Air pollution and hospital admissions for asthma in a tropical city: Kaohsiung, Taiwan. Inhal Toxicol.

[b95-ehp.0901485] Tsai SS, Goggins WB, Chiu HF, Yang CY (2003a). Evidence for an association between air pollution and daily stroke admissions in Kaohsiung, Taiwan. Stroke.

[b96-ehp.0901485] Tsai SS, Huang CH, Goggins WB, Wu TN, Yang CY (2003b). Relationship between air pollution and daily mortality in a tropical city: Kaohsiung, Taiwan. J Toxicol Environ Health A.

[b97-ehp.0901485] Villeneuve PJ, Chen L, Rowe BH, Coates F (2007). Outdoor air pollution and emergency department visits for asthma among children and adults: a case-crossover study in northern Alberta, Canada. Environ Health.

[b98-ehp.0901485] Villeneuve PJ, Chen L, Stieb D, Rowe BH (2006). Associations between outdoor air pollution and emergency department visits for stroke in Edmonton, Canada. Eur J Epidemiol.

[b99-ehp.0901485] Wellenius GA, Bateson TF, Mittleman MA, Schwartz J (2005a). Particulate air pollution and the rate of hospitalization for congestive heart failure among Medicare beneficiaries in Pittsburgh, Pennsylvania. Am J Epidemiol.

[b100-ehp.0901485] Wellenius GA, Schwartz J, Mittleman MA (2005b). Air pollution and hospital admissions for ischemic and hemorrhagic stroke among Medicare beneficiaries. Stroke.

[b101-ehp.0901485] Wellenius GA, Schwartz J, Mittleman MA (2006). Particulate air pollution and hospital admissions for congestive heart failure in seven United Status cities. Am J Cardiol.

[b102-ehp.0901485] Xu X, Zborowski JV, Arena VC, Rager J, Talbott EO (2008). Case-crossover analysis of air pollution and cardiorespiratory hospitalizations: using routinely collected health and environmental data for tracking: science and data. J Public Health Manag Pract.

[b103-ehp.0901485] Yamakazi S, Nitta H, Ono M, Green J, Fukuhara S (2007). Intracerebral haemorrhage associated with hourly concentration of ambient particulate matter: case-crossover analysis. Occup Environ Med.

[b104-ehp.0901485] Yang CY (2008). Air pollution and hospital admissions for congestive heart failure in a subtropical city: Taipei, Taiwan. J Toxicol Environ Health A.

[b105-ehp.0901485] Yang CY, Chang CC, Chuang HY, Tsai SS, Wu TN, Ho CK (2004a). Relationship between air pollution and daily mortality in a subtropical city: Taipei, Taiwan. Environ Int.

[b106-ehp.0901485] Yang CY, Chen CC, Chen CY, Kuo HW (2007). Air pollution and hospital admissions for asthma in a subtropical city: Taipei, Taiwan. J Toxicol Environ Health.

[b107-ehp.0901485] Yang CY, Chen CJ (2007). Air pollution and hospital admissions for chronic obstructive pulmonary disease in a subtropical city: Taipei, Taiwan. J Toxicol Environ Health.

[b108-ehp.0901485] Yang CY, Chen YS, Yang CH, Ho SC (2004b). Relationship between ambient air pollution and hospital admissions for cardiovascular diseases in Kaohsiung, Taiwan. J Toxicol Environ Health A.

[b109-ehp.0901485] Yang CY, Hsieh HJ, Tsai SS, Wu TN, Chiu HF (2006). Correlation between air pollution and postneonatal mortality in a subtropical city: Taipei, Taiwan. J Toxicol Environ Health.

[b110-ehp.0901485] Yang Q, Chen Y, Shi Y, Burnett RT, McGrail KM, Krewski D (2003). Association between ozone and respiratory admissions among children and the elderly in Vancouver, Canada. Inhal Toxicol.

[b111-ehp.0901485] Zanobetti A, Schwartz J (2005). The effect of particulate air pollution on emergency admissions for myocardial infarction: a multicity case-crossover analysis. Environ Health Perspect.

[b112-ehp.0901485] Zanobetti A, Schwartz J (2006). Air pollution and emergency admissions in Boston, MA. J Epidemiol Community Health.

[b113-ehp.0901485] Zeger SL, Dominici F, McDermott A, Samet JM (2008). Mortality in the Medicare population and chronic exposure to fine particulate air pollution in urban centers (2000–2005). Environ Health Perspect.

[b114-ehp.0901485] Zeka A, Zanobetti A, Schwartz J (2005). Short term effects of particulate matter on cause specific mortality: effects of lags and modification by city characteristics. Occup Environ Med.

[b115-ehp.0901485] Zeka A, Zanobetti A, Schwartz J (2006). Individual-level modifiers of the effects of particulate matter on daily mortality. Am J Epidemiol.

